# Supplementary material for: Flight trajectory prediction enabled by time-frequency wavelet transform
Source: Nat Commun. 2023 Aug 29;14:5258. doi: 10.1038/s41467-023-40903-9 (PMC10465572; doi:10.1038/s41467-023-40903-9)
Supplement: Supplementary file 1 — Supplementary Information [file 41467_2023_40903_MOESM1_ESM.pdf]

Supplementary Information

August 3, 2023

Title

Flight trajectory prediction enabled by time-frequency wavelet transform

Author

Zheng Zhang[1], Dongyue Guo[2], Shizhong Zhou[2], Jianwei Zhang[1,2], Yi Lin[1,2,*]

Affiliations

[1]College of Computer Science, Sichuan University, Chengdu, 610065, Sichuan, China
[2]National Key Laboratory of Fundamental Science on Synthetic Vision, Sichuan University, Chengdu, 610065, Sichuan, China
[*]Corresponding Author, yilin@scu.edu.cn

1 Experimental Details

1.1 Dataset Profile

The raw flight trajectories used in this work are collected by multi-source Secondary Surveillance Radar (SSR) and Automatic Dependent Surveillance-Broadcast (ADS-B) from a real-world ATC system in China. Multiple preprocessing steps are conducted to ensure the data quality, including data check, multi-source data parsing, track point and flight registering, multi-source data alignment, data filtering, and fusion. The update interval of the integrated trajectories is 20 seconds. The integrated trajectories are further organized as text files and named by the flight number and archived by dates. In each text file, each line represents one trajectory point for that flight. Each trajectory point consists of 12 attributes split by "|", which are from left to right: timestamp, track number, secondary code, flight number, longitude, latitude, altitude, longitude-direction velocity, latitude-direction velocity, vertical velocity, cleared flight level, and selected altitude. Specifically, the timestamp indicates the hour, minute, and second of the corresponding trajectory point. The longitude and latitude are measured in degrees. The altitude is measured in meters. The velocity is measured in kilometers per hour. All other attributes are strings.

The trajectory data is collected from February 1 to March 17, 2021, and a total of 144,605 trajectories (around 3,200 trajectories used on average per day). The types of aircraft in the trajectory data cover Boeing 737-500, Boeing 737-800, Boeing 737-Max-8, Airbus A319, Airbus A320, etc., which are representative passenger aircraft of civil aviation. As illustrated in Supplementary Fig. 1, the ranges of dynamic attributes are [93.88, 113.68] for the longitude, [19.30, 37.58] for the latitude, [0, 13710] for the altitude, [-945.75, 878.25] for longitude-direction velocity, [-963.50, 925.50] for latitude-direction velocity and [-48.00, 43.00] for vertical velocity.

To construct the experimental dataset, the sliding-window method is employed to generate samples from the complete flight trajectory sequence. For trajectory prediction implemented by using historical M points, the sliding-window size is $M + 1$ and the sliding step is 1. After this process, the longitude, latitude, altitude and velocities for the three corresponding dimensions are selected to formulate the samples. Each sample consists of 10 consecutive trajectory points, where the first 9 points represent the historical trajectory sequence (i.e., model input) and the last one represents the trajectory point to be predicted (i.e., model output). In

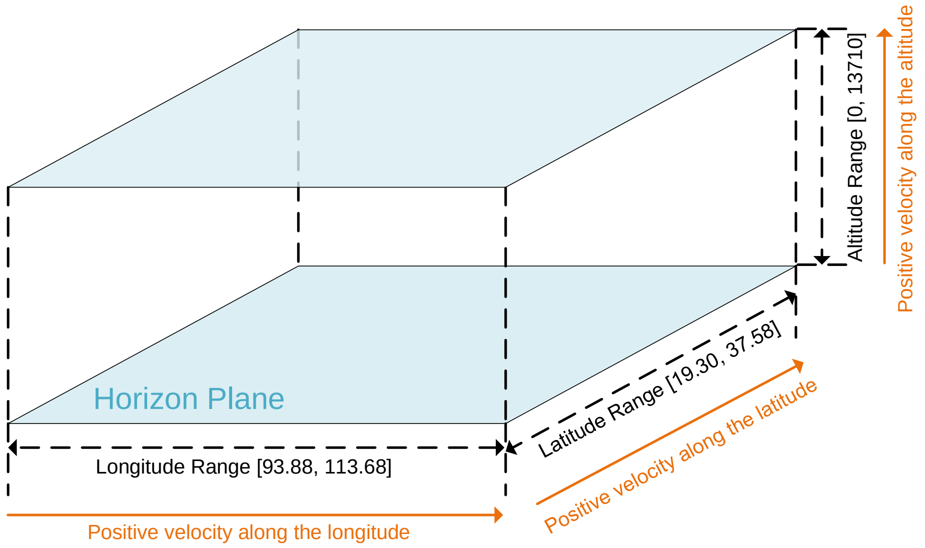

Supplementary Figure 1. The illustration of the coordinate ranges and velocity directions for each dimension. The data ranges of the longitude, latitude and altitude can be represented as a three-dimensional cube. The orange arrows indicate the velocity directions.

Supplementary Table 1. Configurations of the proposed framework.

Arguments	Values
Number of historical trajectory points	9
Dimension of the trajectory embeddings	64
Dimension of the enhanced trajectory embeddings	64
Dimension of the contextual embeddings	64
Layer number of the LSTM block in the encoder	4
Layer number of the LSTM block in the decoder	1
Convolution arguments for 1-level sub-band ([kernel size, stride, padding])	[2, 2, 1]
Convolution arguments for 2 level sub-band ([kernel size, stride, padding])	[3, 3, 0]
Convolution arguments for 3 level sub-band ([kernel size, stride, padding])	[5, 5, 1]
Wavelet basis	Haar
The length of the wavelet filter	2

addition, to eliminate the effects of the measurements and ranges of different attributes, all the six attributes of each sample are normalized by a maximum-minimum method, which can be mathematically illustrated as:

$$a_i^* = \frac{a_i - amin_i}{amax_i - amin_i} \tag{1}$$

where a_i denotes the i-th attribute, and a_i^* serves as its normalized value. $amax_i$ and $amin_i$ are the maximum and the minimum of the i-th attribute. Note that the altitude is first divided by 10 and rounded before being normalized, i.e., the altitude is measured in 10 meters in the dataset.

1.2 Experimental Settings

The proposed framework is trained and tested on a deep-learning platform. The software environment is mainly based on Python 3.7, PyTorch 1.4 and Ubuntu 16.04 operating system. The hardware configurations are listed below: Intel(R) Xeon(TM) E5-2690@2.90GHz processors, 128G of memory, 8TB of hard disks, and eight NVIDIA(R) GeForce RTX(TM) 2080 Ti 11G GPUs. To speed up the training and testing, batch computing techniques were used throughout the experimental process, with a batch size of 2048 and a total number of 150 training epochs. In each training epoch, each batch of samples was randomly selected from the training set without duplicates. During validation and testing, each batch was loaded in the order of text reading. The whole training process lasted for about 4 days with a single GPU. The other baseline models were also trained in the same dataset, hardware and software environment as the proposed framework. For

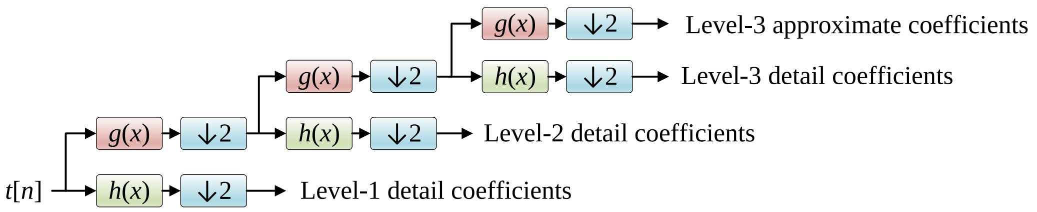

Supplementary Figure 2. The DWT over 3 levels. The g and h are low- and high-pass filters, respectively. $\downarrow 2$ is the downsampling operator.

the model parameters, the detailed configuration list of the proposed framework is shown in Supplementary Table 1.

Specifically, the convolution arguments of the wavelet attention module are presented, with different sub-band argument configurations corresponding to different wavelet components to align the temporal dimension of the wavelet components. Meanwhile, except for the ablation experiments for the wavelet level, the WTFTP framework is performed based on one-level wavelet analysis in all other experiments. To achieve the trajectory prediction task, 9-step historical trajectory points are selected as the input trajectory sequence to infer the trajectory point at the next instant. Based on the 20-second update interval of the dataset, the historical trajectory sequence takes 3 minutes, which suffices to characterize the trend of the aircraft motion state in the short term, as well as to reflect the ongoing maneuvers of the aircraft (i.e., climb, descent, turn, etc.). Therefore, it is believed to feasibly support the experimental analysis of the baseline model and the proposed framework. The same historical trajectory length was also applied for the experiment of other baseline models, with the prediction of the next-instant trajectory point.

In addition, for multi-step prediction, an iterative multi-step (IMS) prediction method is required for all models, i.e., pseudo-labels should be employed as part of the input trajectory to predict longer time steps. Mathematically, the IMS procedure can be shown as:

$$\begin{aligned} \hat{\mathbf{P}}_N &= f(\mathbf{P}_{N-M:N-1}) \\ \hat{\mathbf{P}}_{N+1} &= f([\mathbf{P}_{N-M+1:N-1}, \hat{\mathbf{P}}_N]) \\ &\cdots \\ \hat{\mathbf{P}}_{N+K-1} &= f([\mathbf{P}_{N-M+K-1:N-1}, \hat{\mathbf{P}}_{N:N+K-2}]) \end{aligned} \tag{2}$$

where K denotes the prediction horizon. The definition of other notations follows the main text.

2 Methodology Details

2.1 Preliminary Method - Wavelet Analysis

Wavelet analysis, also known as wavelet transform [1], is a sophisticated signal processing technique to provide feature representations both in time and frequency domains. It usually operates by shifting a scaled wavelet function across the original signal, which enables the extraction of informative time-frequency patterns. The integral form of wavelet transform is defined as the standard inner product of the wavelet function $\psi(\cdot) \in L^2(\mathbb{R})$ and the signal $x(\cdot)$ which can be shown mathematically:

$$C[x](a,b) = \frac{1}{\sqrt{a}} \int_{-\infty}^{+\infty} x(t) \overline{\psi\left(\frac{t-b}{a}\right)} dt \tag{3}$$

where a is the dilation factor, b is the position factor. $C[x](a,b)$ is called wavelet coefficients. The upper line is the conjugate symbol. In general, the dilation factor a is typically inversely proportional to the frequency. The position factor b enables wavelet analysis to capture local frequency information while retaining temporal information. Frequency properties can be obtained within any local periods of time using different combinations of a and b.

In a discrete case, a signal is decomposed through a series of filters and downsampling operators as shown in Fig. 2. This technique, known as discrete wavelet transform (DWT), is based on Multi-Resolution Analysis (MRA, also called multi-scale analysis) [2] and can decompose the original time series into wavelet coefficients at different time and frequency resolutions. The general step of MRA is to divide the signal into two

fundamental components: low-frequency and high-frequency sub-bands. The former called approximated coefficients is obtained by passing the signal through a low-pass filter, while the latter is generated through a high-pass filter and represents detail coefficients. Specifically, an effective procedure of the DWT, i.e. Mallat algorithm [2], can be represented in the mathematical form as follows:

$$a_{j-1,k} = \sum_{n \in \mathbb{Z}} G_{n-2k} a_{j,n} \tag{4}$$

$$d_{j-1,k} = \sum_{n \in \mathbb{Z}} H_{n-2k} a_{j,n} \tag{5}$$

where G is a low-pass filter and H is a high-pass filter. Let V_j be the approximate space generated by the scaled function $\varphi(\cdot)$ with standard orthogonal basis functions $\{2^{j/2}\varphi(2^j t - k)\}_{k \in \mathbb{Z}}$, so $\{a_{j-1,k}\}_{k \in \mathbb{Z}}$ are the approximate coefficients of the original signal $\{a_{j,n}\}_{n \in \mathbb{Z}} \in V_j$ and $\{d_{j-1,k}\}_{k \in \mathbb{Z}}$ are the detail coefficients. To achieve refined time-frequency characteristics, an iterative decomposition of the low-frequency component of each level is performed to formulate a binary-tree structure, where each leaf node denotes a distinct sub-space of time-frequency characteristics. This filter bank is a powerful tool for analyzing signals, images, and other digital data in the wavelet domain. The wavelet coefficients exhibit progressively lower frequency with the increasing of the level of the binary tree. By traversing this tree, the informative features of dynamic details can further be effectively extracted from the original signal. In addition, to recover the time-domain sequence, an inverse operation of the DWT is employed to recursively combine the paired coefficients backward.

2.2 Inference Details

In order to intuitively demonstrate how the proposed WTFTP framework generates wavelet components, further reconstructs the historical trajectory sequence and predicts the next-instant trajectory point, the methodology will be explained in detail with Supplementary Fig. 3. For this purpose, one level of wavelet transform is applied to analyze the trajectory sequence.

As shown in Supplementary Fig. 3a, the historical trajectory sequence is first normalized by the maximum-minimum method and then fed into the neural architecture with the different decoders to generate the wavelet components. Since $L = 1$ level of wavelet analysis is employed, the WTFTP framework is expected to generate $L + 1 = 2$ wavelet components for each trajectory attribute sequence, which are the low- and high-frequency coefficients of level-1 wavelet sub-bands, i.e., WTC_0 and WTC_1, respectively. By the mentioned operations, the WTFTP framework generates a total of $(L + 1) \times d = 12$ ($d = 6$ is the number of attributes) wavelet components, each comprising the dynamic features of the historical trajectory sequence and the next-instant trajectory point, yet these components are in the wavelet domain. Therefore, in the next step, these wavelet components should be operated in inverse discrete wavelet transform (IDWT) procedure to reconstruct the historical trajectory and predict the next-instant trajectory point.

As shown in Supplementary Fig. 3b, the wavelet components of longitude, latitude and altitude are operated by the IDWT procedure. For each attribute, its two wavelet-domain representations, i.e., WTC_0 and WTC_1, are converted by the IDWT to obtain the corresponding time-domain sequence. The time-domain sequence has a time length of 10 that includes the reconstructed values of the historical 9 trajectory points and the predicted value of the next-instant trajectory point, following the trimming of the redundant length due to padding operation. Further, the reconstructed and predicted trajectory can be visualized in the three-dimensional grid after the maximum-minimum denormalization of each trajectory attribute sequence. In such visualization, the blue and orange dots represent the real historical and next-instant trajectory points, respectively. The yellow cross markers represent reconstructed historical trajectory points by the WTFTP framework, while the red cross marker indicates the predicted position at the next instant. Actually, it can be found that there are two optimization tasks of the WTFTP framework, i.e., reconstruction and prediction, by the description of the above inference process, which are designed to explore the correlations between the next-instant trajectory point and the trends or local details of the historical trajectory sequence, that is, in the wavelet domain, the historical and future informative features are inseparable. By performing the IDWT procedure, the characteristics of the next-instant trajectory point can be transformed back to the time domain for supporting the FTP task.

Since the wavelet components of the longitude, latitude and altitude in Supplementary Fig. 3b are represented in the wavelet domain, it would be difficult to understand the trends or details of flight trajectory underlying these components, especially the high-frequency components, WTC_1. To further elaborate the global trends or local details embedded in the wavelet components, as shown in Supplementary Fig. 3c, an alternative IDWT procedure is demonstrated to transform the wavelet components back to the time domain,

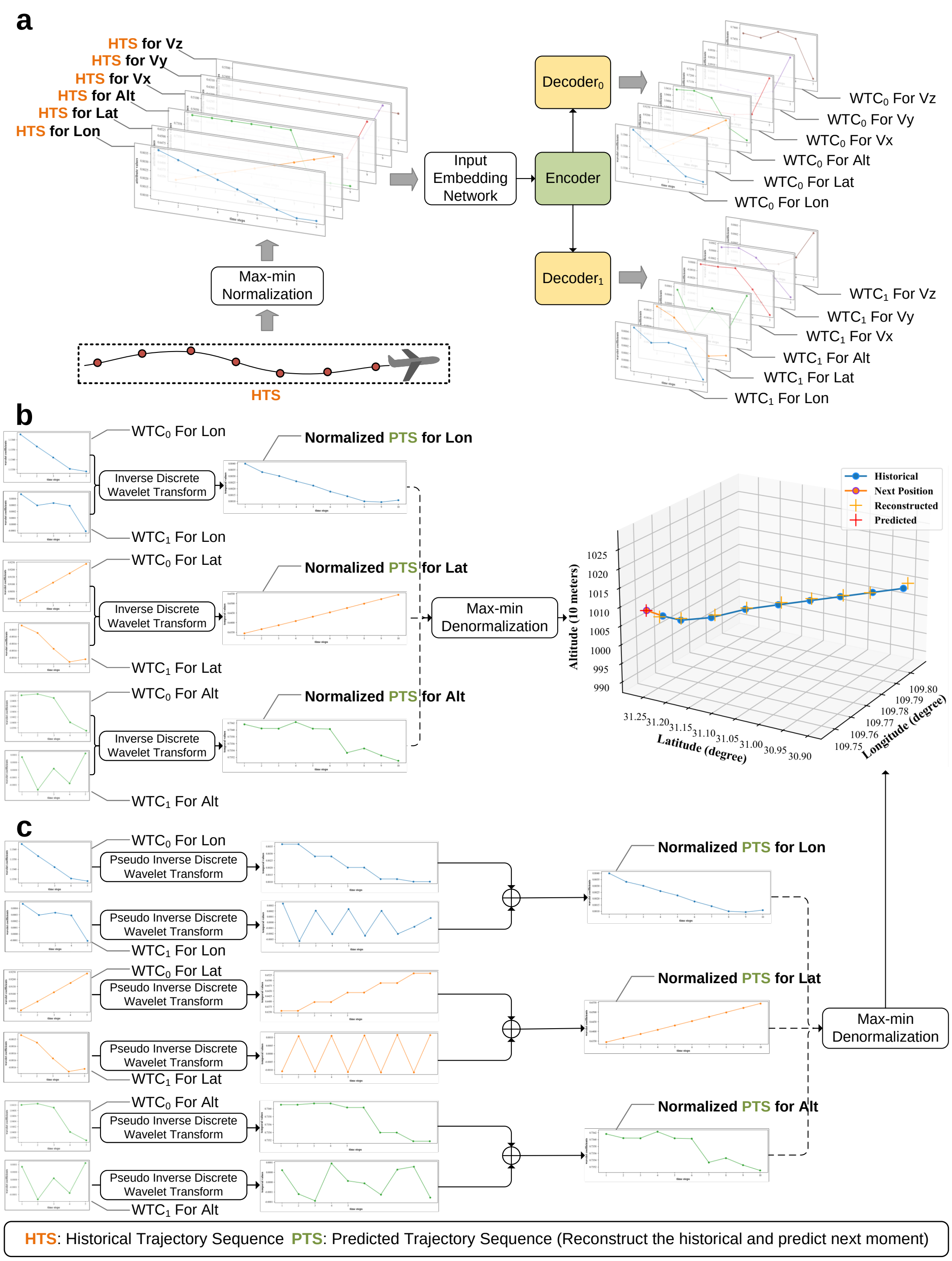

Supplementary Figure 3. Inference details of the proposed framework for one-level wavelet analysis. **a** The historical trajectory sequence is normalized by a maximum-minimum method and fed into the proposed neural architecture. Following the feature extraction of the neural architecture, the final scale-oriented wavelet components are generated by different decoders. **b** By performing the IDWT on the wavelet components of the latitude, longitude and altitude, the time series of the trajectory attributes can be obtained, including the reconstructed historical trajectory and the predicted trajectory point at the next instant. **c** An alternative manner of implementing the IDWT procedure is applied to illustrate the trends and details of the temporal sequence in a more intuitive way.

and thus to visualize and understand different dynamical features represented by them. Unlike the orthodox IDWT procedure indicated in Supplementary Fig. 3b, the two wavelet components of each attribute are sepa-

rately operated by a pseudo-IDWT procedure (i.e., the other sub-band coefficients are set to zero and they are together operated by the orthodox IDWT procedure to obtain the time-domain sequence). In this way, each wavelet component of the flight trajectory attribute illustrates a transformed representation in the time domain, and consequently we can intuitively understand the trends or details underlying them. Taking the longitude as an example, it is found that the time-domain sequence corresponding to WTC_0 can approximately represent the global trends (the longitude sequence gradually decreases, then flattens out and finally rises slightly). Moreover, the time-domain sequence corresponding to WTC_1 indicates the local motions of the aircraft in wave-like forms, i.e., the changes of amplitude reflect the maneuvers states of the aircraft. In particular, as the longitude sequence tends to be flat, the amplitude becomes gradually smaller, while the waveform reflects a phase gap when the longitude sequence rises slightly. Since the IDWT procedure is technically dependent on multiple linear filters, the sum of the different wavelet components through the pseudo-IDWT also results in the time-domain sequence obtained through the orthodox IDWT procedure, i.e., the reconstructed values of the historical 9 trajectory points and the predicted value of the next-instant trajectory point.

3 Multi-Step Prediction Analysis

In air traffic research, it is believed that the multi-step prediction for the flight trajectory can enhance the applicability of the proposed model by considering the future traffic situation with higher predictability. In general, the proposed WTFTP framework is to achieve the trajectory prediction task by reconstructing the historical trajectory sequence and predicting the trajectory attributes in the next time instant based on the wavelet coefficients generated by the neural architecture. Therefore, in this work, the iterative prediction procedure is performed to achieve the multi-step prediction as illustrated in Supplementary Equation (2), i.e., using previously predicted values as pseudo-labels for the prediction of longer time steps. Meanwhile, to formulate a comprehensive performance comparison, other comparative baselines in the main text are also applied to estimate the flight trajectories in multiple time steps, in which all baselines conduct the IMS prediction.

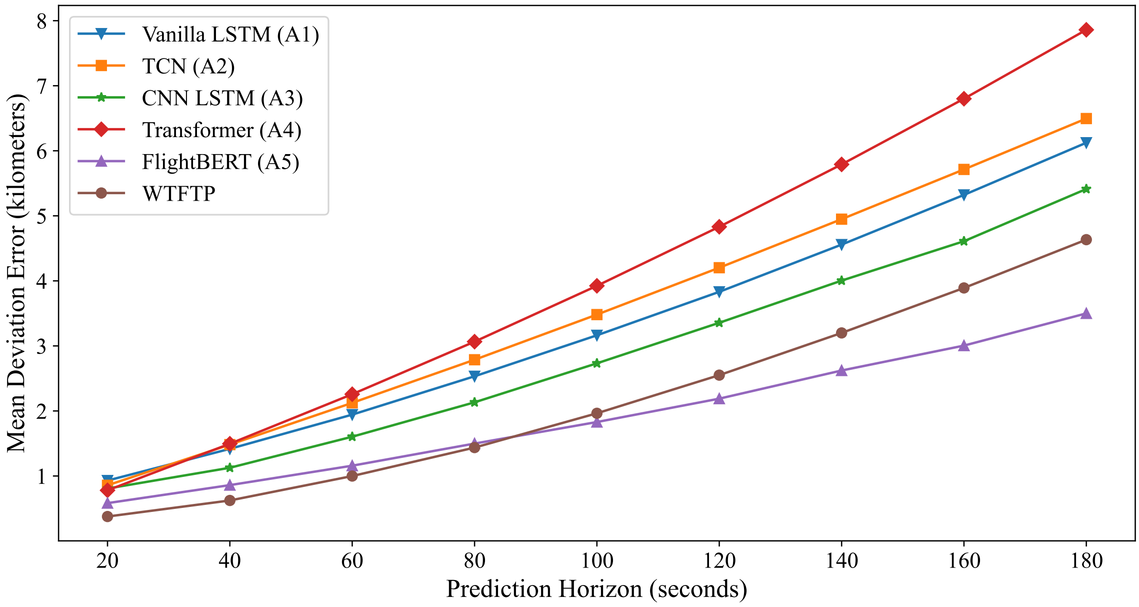

Supplementary Figure 4. Mean deviation error of the WTFTP framework and baselines at different prediction horizons. The maximum horizon is 180 seconds, i.e., the 9-step ahead IMS prediction. The notation of each baseline is presented in brackets in the figure legend.

The experimental results are shown in Supplementary Fig. 4, where the multi-step prediction performance obtained by all the comparative baselines is measured in terms of the mean deviation error (MDE) in kilometers. The calculation details of the MDE can be found in the main text. In addition, the x-axis is organized by the seconds to practically support the air traffic control applications. The second-measured MDE can indicate the predictability of the future traffic situation, which allows the air traffic controllers to evaluate traffic safety in advance. Based on the update interval of the flight trajectory dataset (20 seconds),

the measurements in the x-axis can be mapped to 1-10 steps.

In general, as can be seen from Supplementary Fig. 4, the prediction performance in terms of the MDE gradually rises as the prediction horizon increase, which is compatible with the inherent mechanism of the IMS prediction procedure. Among selective baselines, the multi-step prediction presents quite different performance from that of the single-step prediction. Specifically, the Transformer model (A4) suffers from the poorest multi-step prediction performance. As discussed in Dlinear [3], the self-attention mechanism is permutation-invariant. Since the ordering information of the temporal sequence is provided by positional embeddings, it inevitably results in the temporal information loss for multi-step inference. Therefore, the Transformer model without well-designed techniques (e.g. temporal modeling, feature representation, etc.) fails to obtain comparable multi-step prediction performance, even though its desired single-step performance. Although the FlightBERT model (A5) is also constructed based on Transformer blocks, the targeting technical improvements, including binary encoding, the attribute correlation attention block, as well as the hybird constrained loss function, bridge the performance gaps and achieve desired multi-step prediction performance. For the TCN model (A2), although the causal convolution is employed to ensure the unidirectional flow of the temporal features, as that of the self-attention mechanism, the ordering information of the temporal sequence is inevitably corrupted to degrade multi-step prediction performance, i.e., only better than that of A4. As to the RNN-based models, i.e., the vanilla LSTM (A1) and the CNN LSTM model (A3), they yield better multi-step prediction performance compared to A2 and A4. As a common deep-learning model in existing FTP methods, the vanilla LSTM (A1) is able to employ the gated mechanism and recurrent computing architecture to capture long- and short-term dynamics, supporting the multi-step prediction. Despite the impact of accumulated errors, it is evident that A1 provides a relatively accurate estimation of future flight trend thanks to the recurrent inference on the historical trajectory points. Based on this, A3 further enhances the performance of downstream recurrent inference by allowing the extraction of local spatial features using convolutional filters.

As stated in this work, benefiting from the time-frequency analysis based on wavelet transform, the WTFTP framework is able to achieve fine-grained modeling of local motion details. However, it is also the fine-grained modeling of local motion details that leads to performance degradation in the IMS prediction at longer time steps since the WTFTP framework is susceptible to anomalies in the historical trajectory sequence (i.e., over-modeling the sequential changes caused by using previously predicted values as pseudo-labels). Nevertheless, it is evident that the proposed WTFTP framework still outperforms baselines A1-A4 within 180 seconds. As to the results for the proposed WTFTP framework and the FlightBERT model, the proposed WTFTP framework has the ability to achieve prominent performance improvement over the FlightBERT model within 80 seconds due to the modeling ability to global flight trends and local motion details by time-frequency analysis. However, with the accumulation of the iterative prediction errors, it fails to obtain comparable performance beyond the 5th prediction step (100 seconds ahead). As illustrated in the main text, the results also inspire us to implement a time-frequency analysis-based multi-step prediction framework in future works, which is expected to practically support the air traffic operations.

References

[1] Mallat Stéphane. *A Wavelet Tour of Signal Processing (Third Edition)*. Academic Press, Boston, 2009.

[2] Mallat Stéphane. A theory for multiresolution signal decomposition: the wavelet representation. *IEEE Transactions on Pattern Analysis and Machine Intelligence*, 11(7):674–693, 1989.

[3] Ailing Zeng, Muxi Chen, Lei Zhang, and Qiang Xu. Are transformers effective for time series forecasting? *arXiv preprint arXiv:2205.13504*, 2022.
