# Supplementary material for: Flight trajectory prediction enabled by time-frequency wavelet transform
Source: Nat Commun. 2023 Aug 29;14:5258. doi: 10.1038/s41467-023-40903-9 (PMC10465572; doi:10.1038/s41467-023-40903-9)
Supplement: Supplementary file 3 — Reporting Summary [file 41467_2023_40903_MOESM3_ESM.pdf]

nature portfolio

Corresponding author(s): Yi Lin

Last updated by author(s): Aug 12, 2023

Reporting Summary

Nature Portfolio wishes to improve the reproducibility of the work that we publish. This form provides structure for consistency and transparency in reporting. For further information on Nature Portfolio policies, see our Editorial Policies and the Editorial Policy Checklist.

Statistics

For all statistical analyses, confirm that the following items are present in the figure legend, table legend, main text, or Methods section.

n/a	Confirmed	
☐	☒	The exact sample size (*n*) for each experimental group/condition, given as a discrete number and unit of measurement
☐	☒	A statement on whether measurements were taken from distinct samples or whether the same sample was measured repeatedly
☒	☐	The statistical test(s) used AND whether they are one- or two-sided<br>*Only common tests should be described solely by name; describe more complex techniques in the Methods section.*
☒	☐	A description of all covariates tested
☒	☐	A description of any assumptions or corrections, such as tests of normality and adjustment for multiple comparisons
☐	☒	A full description of the statistical parameters including central tendency (e.g. means) or other basic estimates (e.g. regression coefficient) AND variation (e.g. standard deviation) or associated estimates of uncertainty (e.g. confidence intervals)
☒	☐	For null hypothesis testing, the test statistic (e.g. *F*, *t*, *r*) with confidence intervals, effect sizes, degrees of freedom and *P* value noted<br>*Give P values as exact values whenever suitable.*
☒	☐	For Bayesian analysis, information on the choice of priors and Markov chain Monte Carlo settings
☒	☐	For hierarchical and complex designs, identification of the appropriate level for tests and full reporting of outcomes
☒	☐	Estimates of effect sizes (e.g. Cohen's *d*, Pearson's *r*), indicating how they were calculated

Our web collection on statistics for biologists contains articles on many of the points above.

Software and code

Policy information about availability of computer code

Data collection	No software was used.
Data analysis	No software was used.

For manuscripts utilizing custom algorithms or software that are central to the research but not yet described in published literature, software must be made available to editors and reviewers. We strongly encourage code deposition in a community repository (e.g. GitHub). See the Nature Portfolio guidelines for submitting code & software for further information.

Data

Policy information about availability of data

All manuscripts must include a data availability statement. This statement should provide the following information, where applicable:

- Accession codes, unique identifiers, or web links for publicly available datasets
- A description of any restrictions on data availability
- For clinical datasets or third party data, please ensure that the statement adheres to our policy

We are not authorized to publicly release the whole dataset used during the current study concerning safety-critical issues. Nonetheless, the processed example samples are available on https://zenodo.org/record/8238768 (ref.[61]). Source data for all figures, except Figure 3, are provided as Source data file. The trajectory sequence in Figure 3 spans a wide range of locations, thus it cannot be made publicly available for the safety of China civil aviation.

Research involving human participants, their data, or biological material

Policy information about studies with human participants or human data. See also policy information about sex, gender (identity/presentation), and sexual orientation and race, ethnicity and racism.

Reporting on sex and gender	N/A
Reporting on race, ethnicity, or other socially relevant groupings	N/A
Population characteristics	N/A
Recruitment	N/A
Ethics oversight	N/A

Note that full information on the approval of the study protocol must also be provided in the manuscript.

Field-specific reporting

Please select the one below that is the best fit for your research. If you are not sure, read the appropriate sections before making your selection.

☐ Life sciences ☐ Behavioural & social sciences ☒ Ecological, evolutionary & environmental sciences

For a reference copy of the document with all sections, see nature.com/documents/nr-reporting-summary-flat.pdf

Ecological, evolutionary & environmental sciences study design

All studies must disclose on these points even when the disclosure is negative.

Study description	The primary goal of this study is to predict primary motion attributes that describe discrete trajectory points of aircraft, such as longitude, latitude, altitude. Four metrics are applied to measure prediction performance and 45-day trajectory dataset serves as experimental data source.
Research sample	Each trajectory sample is parsed from raw binary files collected from a real ATC system and consists of six attributes, i.e., longitude, latitude, altitude and speed along previous position components.
Sampling strategy	The sample size in training and test procedures is 2048, i.e., batch size for tensor calculating. This is a trade-off between time cost for training or inferring and memory limit of the GPU accelerator.
Data collection	The raw flight trajectories are collected by multi-source Secondary Surveillance Radar (SSR) and Automatic Dependent Surveillance-Broadcast (ADS-B) from a real-world ATC system in China.
Timing and spatial scale	The 45-day raw trajectory data is collected from February 1 to March 17, 2021. The update period is 20 seconds. The range of the longitude and latitude are [93.88, 113.68] and [19.30, 37.58].
Data exclusions	No data exclusion. Because the flight trajectory dataset has been formulated by multiple preprocessing steps before, like data check, multi-source data parsing, etc.
Reproducibility	All attempts to repeat the experiment were successful.
Randomization	For training procedure, each batch of samples are selected randomly from the training dataset to perform stochastic gradient descent. For validation and test procedures, the samples are organized by the order of file read, rather than a randomized order.
Blinding	Blinding is not relevant to this study. Because the sample labels involved in this study are the trajectory sequences themselves, and there are no artificially labeled groups. The experimental process has ensured that the training set, the validation set and the test set do not overlap, and thus there is no artificial factors in the experimental results.

Did the study involve field work? ☐ Yes ☒ No

Reporting for specific materials, systems and methods

We require information from authors about some types of materials, experimental systems and methods used in many studies. Here, indicate whether each material, system or method listed is relevant to your study. If you are not sure if a list item applies to your research, read the appropriate section before selecting a response.

Materials & experimental systems

n/a	Involved in the study
☒	☐ Antibodies
☒	☐ Eukaryotic cell lines
☒	☐ Palaeontology and archaeology
☒	☐ Animals and other organisms
☒	☐ Clinical data
☒	☐ Dual use research of concern
☒	☐ Plants

Methods

n/a	Involved in the study
☒	☐ ChIP-seq
☒	☐ Flow cytometry
☒	☐ MRI-based neuroimaging
